# Neurodevelopmental disorders and anti-epileptic treatment in a patient with a *SATB1* mutation: A case report

**DOI:** 10.3389/fped.2022.931667

**Published:** 2022-09-02

**Authors:** Ying Yu, Cuiyun Li, Wei Li, Liting Chen, Dan Wang, Jie Wang, Jian Wang, Ruen Yao

**Affiliations:** ^1^Antenatal Diagnostic Center, Sanya Women and Children’s Hospital Managed by Shanghai Children’s Medical Center, Sanya, China; ^2^Department of Science and Education, Sanya Women and Children’s Hospital Managed by Shanghai Children’s Medical Center, Sanya, China; ^3^Molecular Genetic Diagnosis Center, Sanya Women and Children’s Hospital Managed by Shanghai Children’s Medical Center, Sanya, China; ^4^Molecular Diagnostic Laboratory, Department of Medical Genetics, Shanghai Children’s Medical Center, School of Medicine, Shanghai Jiao Tong University, Shanghai, China

**Keywords:** *SATB1*, epilepsy, anti-epileptic drugs, neurodevelopmental delay, protein-truncating variants

## Abstract

SATB1 variants causing developmental delay with dysmorphic facies and dental anomalies have been reported in a small cohort. Most patients present epilepsy as a main clinical feature in neurodevelopmental disorders; however, its treatment is unknown. Here, we present a Chinese patient with a *de novo* truncating variation in *SATB1* who presented with mild developmental delay. We disclose the detailed anti-epileptic pharmacological treatment that enabled a favorable outcome. Our study provides important information that may aid clinicians in the prognosis and treatment of rare neurological developmental disorders caused by gene mutations.

## Introduction

Variants of *SATB1* cause clinically overlapping but distinct neurodevelopmental disorders, including intellectual disability, muscle tone abnormalities, hypotonia, spasticity, epilepsy, behavioral problems, facial dysmorphisms, and dental abnormalities. Genotype-phenotype relationships associated with each pathophysiological mechanism have been identified (1). In the limited number of reported clinical cases, motor and speech delays have been the most prevalent neurological manifestations (92 and 89%, respectively). Epilepsy accounts for 61% of all reported nervous system-related phenotypes and is the only symptom with a relatively comprehensive pharmacological treatment. Studies on most neurodevelopmental disorders have proved that the control of early epilepsy is crucial for slowing the progression of neurological impairment and restoring normal neurological function (2, 3). Details of the specific epileptic condition and therapeutic interventions in patients are not available in existing reports of *SATB1* variants, thus lacking the clinicians’ and patients’ perspectives of the treatment experience. This study reports a Chinese patient with a pathogenic *SATB1* mutation manifested as epilepsy, growth retardation, and facial dysmorphisms. The treatment process of epilepsy and the growth and development history of the patient are elaborated. Our study provides relevant clinical and pharmacological information for the prognosis and treatment of neurological rare developmental disorders caused by gene mutations.

## Case description

The proband was a 7-year-old girl. She was referred to the Medical Genetics Clinic of Shanghai Children’s Medical Center, Sanya Women and Children’s Hospital, presenting with global developmental delay. She was the only child of non-consanguineous Han Chinese parents. The patient had no abnormalities on prenatal examination and was born naturally at full term. Her parents were physically healthy and had no relevant family history.

**FIGURE 1:**
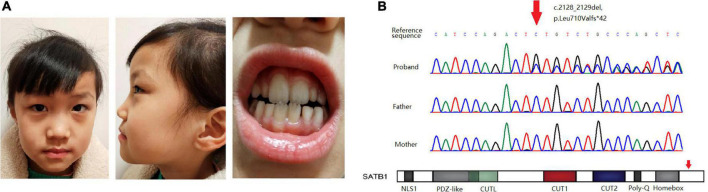
**(A)** Facial features and dental abnormalities are not recognizable in the patient. **(B)** Sanger sequencing of the variant in the pedigree and location of the variant in the gene.

**FIGURE 2:**
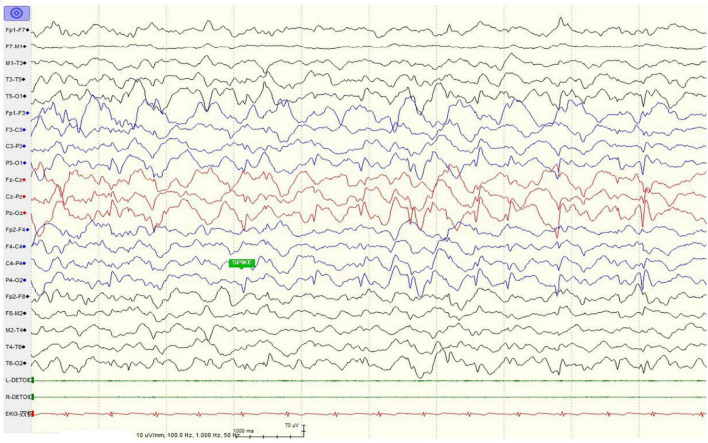
Electroencephalogram of the patient showed only scarce sharp waves during sleep at age seven after anti-epileptic drug treatment.

The girl sought medical advice for the first time at age 2. She began to roll her eyes up and frequently stopped moving for several seconds, mostly on stimulation. In 2 months, she had a major seizure that lasted approximately 5 min, with convulsions affecting her whole body, her eyes rolling up, and her mouth foaming. Facial features were not recognizable, and dental abnormalities were not evident (Figure 1A). She was diagnosed with epilepsy at the local hospital. The EEG (Electroencephalography) showed sharp, sharp-slow, and spinous slow waves in the bilateral occipital areas, but were more prominent on the right side. There were more asynchronous, sharp, slow, and spike waves in the bilateral frontal, middle, and central regions. The patient started anti-epileptic drug therapy. Valproic acid was used for 2 months at a dose of 250 mg (25 mg/kg) twice daily. Seizures occurred several times during this period, and EEG did not improve significantly. For the following 9 months, the dose of valproic acid was reduced to 100 mg (10 mg/kg), and 80 mg oxcarbazepine (8 mg/kg) was introduced, both administered twice daily for better control of the epileptic activity. With the increase in body weight, the doses of valproic acid and oxcarbazepine were adjusted. At the age of seven, when this case was reported to us, the regimen consisted of 160 mg valproic acid (9 mg/kg) and 180 mg oxcarbazepine (10 mg/kg) twice daily. Epileptic activity had been well controlled since the combined use of valproic acid and oxcarbazepine. No seizures or other epileptic activity were noticed, and the electroencephalogram at the age of seven showed only scarce sharp waves during sleep (Figure 2).

The Wechsler Intelligence Scale for Children, Fourth Edition (WISC-IV) was used to evaluate the general intellectual ability of the patient when she was 78 months old. The full-scale intelligence quotient was 78, which is at the lower limit of the normal range. The scores of verbal comprehension, perceptual reasoning, working memory, and processing speed subtests were 81, 79, 85, and 79, respectively. Detailed neurological features are shown in Table 1.

**TABLE 1:** Neurological clinical features of patients with SATB1 gene variants.

	Our patient	Previously reported patients by den Hoed J, et al. (1)	
		Null variants (1)	Missense variants
Intellectual disability	Yes	9/10	20/22
Developmental delay	Yes	12/12	23/24
Motor delay	Yes	11/12	23/25
Speech delay	Yes	10/12	22/24
Dysarthria	No	1/10	5/9
Epilepsy	Yes	2/10	20/26
Hypotonia	No	5/12	23/25
Spasticity	No	0/12	10/24
Ataxia	No	2/10	4/14
Behavioral disturbances	Yes (anxiety, mild ASD-features)	7/12	17/22
Sleep disturbances	No	3/11	9/18
Brain imaging abnormalities	No	2/7	17/24
Regression	No	1/12	5/24
Facial dysmorphisms	Yes (subtle)	7/11	17/24
Dental/oral abnormalities	Yes (widely spaced teeth)	6/11	11/24

To detect disease-causing mutations, genomic DNA was extracted from the peripheral blood samples of the patient and her parents using the Gentra Puregene Blood Kit (Qiagen, Hilden, Germany), according to the manufacturer’s protocol. Whole-exome capture was performed using an Agilent SureSelect V6 enrichment capture kit (Agilent Technologies, Inc., Woburn, MA, United States), according to the manufacturer’s instructions. The captured library was sequenced using the Illumina HiSeq 2500 System (Illumina, Inc., San Diego, CA, United States). Original sequencing data were assessed using FastQC (version 0.11.2) for quality control. The Burrows Wheeler alignment tool v0.2.10 was used for sequencing data alignment to the Human Reference Genome (NCBI build 37, hg 19). Single-nucleotide variants and small indels were identified using the Genome Analysis Toolkit. All variants were saved in VCF format and uploaded to the Ingenuity Variant Analysis (Ingenuity Systems, Redwood City, CA, United States) and TGex (Translational Genomics Expert) platforms for biological analysis and interpretation, as previously reported (4). Variants detected by next-generation sequencing were confirmed by Sanger sequencing.

Whole-exome sequencing revealed a novel frame-shift variant, c.2128_2129del, p.Leu710Valfs × 42, in the last exon of *SATB1* (NM_001195470.2). Sanger sequencing confirmed the variant as well as the wild-type status of her father and mother (Figure 1B). The control population database (gnomAD) and our local control cohort database did not have reports on the detected variant. The frame-shift variant is classified as a pathogenic variant according to the ACMG guidelines for variant interpretation.

## Discussion

*SATB1* (OMIM × 602075) encodes a transcription factor involved in T cell development and maturation (5). The pathogenicity of *SATB1* variants was initially reported upon the identification of *de novo* variants in two large neurodevelopmental disorder cohorts. These variants suggested a role for this gene in neurodevelopment (6, 7). Accurate genotype-phenotype correlations and disease mechanisms were recently identified during the clinical evaluation of a 42-patient cohort (1). Although the broad phenotypic spectrum of *SATB1* mutations has been described (including neurodevelopmental delay, intellectual disability, muscle tone abnormalities, epilepsy, behavioral problems, facial dysmorphisms, and dental abnormalities), the treatment and prognosis of these patients have not been reported previously.

Epilepsy is the only disease symptom for which systematic drug regimens are available, as more than 25 antiseizure medications are currently used. The association between epilepsy and neurodevelopmental disorders is well established. Children, especially newborns, infants, and young children, with developmental epilepsies have an increased risk of cognitive, neurobehavioral, and psychiatric disorders (8–10). This has important implications for treatment, especially in children with epileptic encephalopathy, in whom early and successful treatment of seizures and interictal epileptiform activity may be crucial for positive neurodevelopmental outcomes (11, 12). In children with *SCN1A* seizure disorders, who are at a high risk of sudden unexplained death in epilepsy, seizure control is critical. In addition, prolonged acute seizures may cause permanent injury. In Dravet syndrome, cognitive deterioration may occur, especially when seizure control is incomplete (3, 13). A beneficial effect of immunotherapy combined with anti-epileptic drugs on seizure frequency and cognition has been observed in patients (14). A strong link between seizure control and improvement in neurological function has been observed in many common and rare epilepsy syndromes (15, 16). The combination of low-dose valproic acid and oxcarbazepine showed satisfactory effects on the control of epilepsy in our patient; her neurodevelopment also improved since she was seizure-free. Satb1 was expressed in midbrain dopaminergic neurons and acted as a dopaminergic neuron-specific regulator (17, 18). The attenuation effect of dopaminergic neurotoxicity by valproic acid was assumed to be effective (19). Oxcarbazepine might effectively reduce seizure frequency when used as an add-on for drug-resistant epilepsy (20), but the mechanism by which valproic acid and oxcarbazepine combination works in patients with SATB1 variants needs further investigation. Treatment process and prognosis of our case could provide reliable reference for management of patients with same gene mutation.

A clear genotype-phenotype correlation has been observed: individuals carrying missense variants were more severely affected than individuals carrying protein-truncating variants (1). Functional assays using cells expressing pathogenic variants of SATB1 harboring missense mutations in the CUT1 and CUT2 DNA-binding domains demonstrated altered transcriptional activity compared with the wild-type protein, which could explain this genotype-phenotype correlation (1). Although the *de novo* frame-shift variant detected in our patients was located in the last exon of the gene, it is likely to escape nonsense-mediated mRNA decay, as a variant located at a more distal position has been reported (p.N736fs × 8). Our patient showed a mild phenotype consistent with previously reported patients presenting protein-truncating variants as a distinct group given the haploinsufficiency of these mutations (Table 1). Diagnosis in patients with mild to moderate developmental delay without evident facial dysmorphism and dental/oral abnormalities is difficult; thus, genetic testing in these patients is quite effective for diagnostic purposes.

## Conclusion

We report a *de novo* protein-truncating variant of *SATB1* in a Chinese patient with epilepsy, developmental delay, and dysmorphic features. The clinical features of our patient are consistent with previously reported genotype-phenotype correlations. The anti-epileptic drug treatment of the patient is described in detail, providing important information for the control of epilepsy in patients with *SATB1* variations, which is crucial for their neurodevelopment.

## Data availability statement

The data presented in this study are deposited in the GSA for human repository, accession number SubHRA003891.

## Ethics statement

The studies involving human participants were reviewed and approved by Sanya Women and Children’s Hospital Managed by Shanghai Children’s Medical Center. Written informed consent to participate in this study was provided by the participants’ legal guardian/next of kin.

## Author contributions

YY, CL, JeW, and RY contributed to the conception and design of the study. CL and WL organized the database. YY and DW performed the statistical analysis. RY and YY wrote the first draft of the manuscript. CL, JaW, and LC wrote sections of the manuscript. All authors contributed to the manuscript revision and read and approved the submitted version.
